# Use of Heavy Water (D_**2**_O) in Developing Thermostable Recombinant p26 Protein Based Enzyme-Linked Immunosorbent Assay for Serodiagnosis of Equine Infectious Anemia Virus Infection

**DOI:** 10.1155/2014/620906

**Published:** 2014-01-09

**Authors:** Harisankar Singha, Sachin K. Goyal, Praveen Malik, Raj K. Singh

**Affiliations:** Veterinary Type Culture Collection, National Research Centre on Equines, Sirsa Road, Hisar, Haryana 125 001, India

## Abstract

Thermostabilizing effect of heavy water (D_2_O) or deuterium oxide has been demonstrated previously on several enzymes and vaccines like oral poliovirus vaccine and influenza virus vaccine. In view of the above observations, effect of heavy water on *in situ* thermostabilization of recombinant p26 protein on enzyme-linked immunosorbent assay (ELISA) for serodiagnosis of equine infectious anemia virus (EIAV) infection was investigated in the present study. The carbonate-bicarbonate coating buffer was prepared in 60% and 80% D_2_O for coating the p26 protein in 96-well ELISA plate and thermal stability was examined at 4°C, 37°C, 42°C, and 45°C over a storage time from 2 weeks to 10 months. A set of positive serum (*n* = 12) consisting of strong, medium, and weak titer strength (4 samples in each category) and negative serum (*n* = 30) were assessed in ELISA during the study period. At each time point, ELISA results were compared with fresh plate to assess thermal protective effect of D_2_O. Gradual increase in the stabilizing effect of 80% D_2_O at elevated temperature (37°C < 42°C < 45°C) was observed. The 80% D_2_O provides the thermal protection to rp26 protein in ELISA plate up to 2 months of incubation at 45°C. The findings of the present study have the future implication of adopting cost effective strategies for generating more heat tolerable ELISA reagents with extended shelf life.

## 1. Introduction

Preserving quality of the biological macromolecules like vaccine, enzymes, antigens, antibody, and so forth is one of most important but difficult tasks as potency and stability of biological molecules are lost in a temperature and time-dependent fashion. Maintaining strict cold chain during manufacture, storage, transportation, and field utilization of these biological macromolecules is necessary for getting optimal effect. Harsh field environment due to high ambient temperature and extensive power shortage are going to make the task more challenging in the future. Various formulation strategies to enhance the temperature stability of vaccines and adjuvant are being explored in different laboratories across the globe. Among these methods, generic expression of recombinant vaccine candidates fused with heat stable protein [1, 2] and heterologous expression of protein in maize seeds [3] have been proved to improve solubility and thermostability of recombinant proteins. Efficacy of chemical stabilizers lactalbumin hydrolysate-sucrose on the thermostability of a live-attenuated *peste des petits ruminants* (PPR) vaccine had previously been demonstrated [4, 5]. Role of heavy water (D_2_O) in biological sciences especially in thermostabilization of vaccines has been thoroughly reviewed [6]. The first experimental evidence of D_2_O mediated thermostabilization was demonstrated in yellow fever 17D vaccines [7]. Later, the potential of D_2_O was extensively studied for thermostabilization of oral polio virus vaccine [8–11]. Subsequently, many candidate biomolecules including influenza virus vaccine [12], *peste des petits ruminants* vaccine [13], haemorrhagic septicaemia vaccine [14], and enzymes like lactate dehydrogenase, hexokinase, and creatine kinase [15] were used to investigate the thermostabilizing effect of D_2_O.

Thorough search and scan of the literature revealed that most of the thermostabilization research was focused on vaccines. Till date, no information on thermostabilization of proteins using D_2_O in enzyme-linked immunosorbent assay (ELISA) is available. However, stabilization of proteins with synthetic stabilizers and molecular chaperons has been reported [16, 17]. Because of inherent problem of limited shelf life of commercial diagnostic reagents especially protein and antibody at temperature higher than recommended storage temperature, enhancing protein stability at high temperature is becoming increasingly demanded. In the present study, the protective thermostable effect of D_2_O on recombinant p26 protein (rp26) in ELISA for diagnosis of equine infectious anemia (EIA) was investigated with the aim of developing thermostable ELISA.

## 2. Materials and Methods

### 2.1. Serum Panel

To evaluate the test performance, sets of reference serum and field serum maintained in the serum repository of NRCE were used. Serum samples (*n* = 12) positive for EIAV antibodies were used as positive control which included seven reference positive (NVSL Ames, IA, USA; VMRD, Pullman, WA, USA; and IDEXX, Westbrook, USA) and five EIAV-infected equine serums collected in routine diagnosis process under the institute's serosurveillance programme.

According to the anti-p26 antibody titer strength, positive serum samples were designated as strong (*n* = 4), medium (*n* = 4), and weak positive (*n* = 4) serums. A panel of 30 known negative serum samples from NRCE repository and reference negative control (*n* = 2) serum (VMRD, Pullman, WA, USA) was also included in the assay.

### 2.2. Indirect ELISA Using Heavy Water (D_2_O)

Indirect ELISA using recombinant EIAV p26 protein was used as described previously [18]. The rp26 (200 ng/well) was coated in 96-well ELISA plate (Greiner Bio One, USA) using standard carbonate-bicarbonate coating buffer (Sigma-Aldrich, St. Louis, USA) prepared in H_2_O, 60% D_2_O (v/v), or 80% D_2_O (v/v). After blocking the plates with 6% skim milk in PBS-T (200 *μ*L/well) for 1 h at 37°C, plates were washed twice with PBS-T. Total 48 plates were coated with each buffer and labeled, and 12 plates from each buffer were incubated at four temperatures (4°C, 37°C, 42°C, and 45°C) for developing at defined time interval (from 2 weeks to 10 months).

To determine the thermostable effect of heavy water, fresh plate was coated with rp26 protein with standard coating buffer and developed along with experimental plates at each time point following the methods described earlier. Serum samples were tested in duplicate. The whole experiment was repeated twice over a period of 2 years.

### 2.3. Data Analysis

ELISA results obtained in each time interval were compared with fresh rp26 coated plate. The absorbance data (OD values) was normalized to eliminate interplate variations and make data comparable across the plates using the following formula: “percent positivity (*PP*%) = (OD_492_  sample  serum − OD_492_  negative  control)/(OD_492_  positive  control − OD_492_  negative  control) × 100%”. The optimum cut-off value of the ELISA was determined by receiver operative curves (ROC) analysis using *PP* value [19]. Thermostable effect of heavy water on rp26 in different temperature was determined as a function of *PP* value. Comparative analysis of *PP* value and statistical significance was determined by Student's *t*-test.

## 3. Results

### 3.1. Evaluation of Thermostable Effect of D_2_O on rp26 ELISA at Different Storage Time and Incubation Temperature

**Figure 1:**
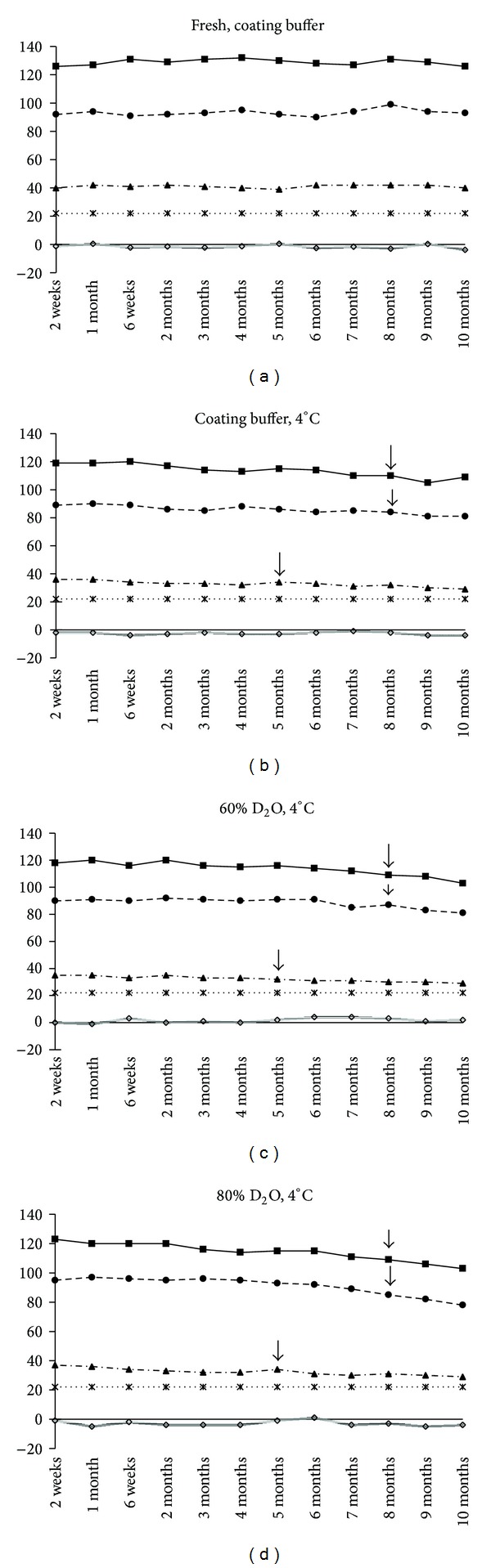
Thermostability of rp26 ELISA at 4°C. *PP* value of strong positive serum (■), medium positive serum (●), and weak positive serum (▲) was compared with fresh ELISA. Duration of thermostability is indicated by arrow (↓) against each serum. Beyond this point, significant deviation in *PP* value was observed in comparison to fresh plate (*P* = 0.03). No significant difference exists in 80% D_2_O versus H_2_O coating buffer, 60% D_2_O versus H_2_O coating buffer, and 60% D_2_O versus 80% D_2_O.

In fresh plate, average *PP*% value for strong, medium, and weak positive serums was 128, 93, and 41 throughout the study period. Cut-off *PP* value of the ELISA was 22 as determined earlier [18]. At 4°C, *PP* value of strong positive serum obtained with coating buffer either prepared in H_2_O- or 60%, and 80% D_2_O-ranged from 121 at 2 weeks to 103 at 10 months. Up to 8 months, the *PP* value (111) of strong positive serum in these buffers did not vary significantly with fresh plate ELISA (*P* = 0.25 − 0.06) (Figure 1). For medium positive serum, the *PP* value gradually decreases from 91 to 78, while for weak positive serum, the value ranged from 37 to 29 during the observation period. The *PP* value obtained with all the serums was above the cut-off point during the study period.

**Figure 2:**
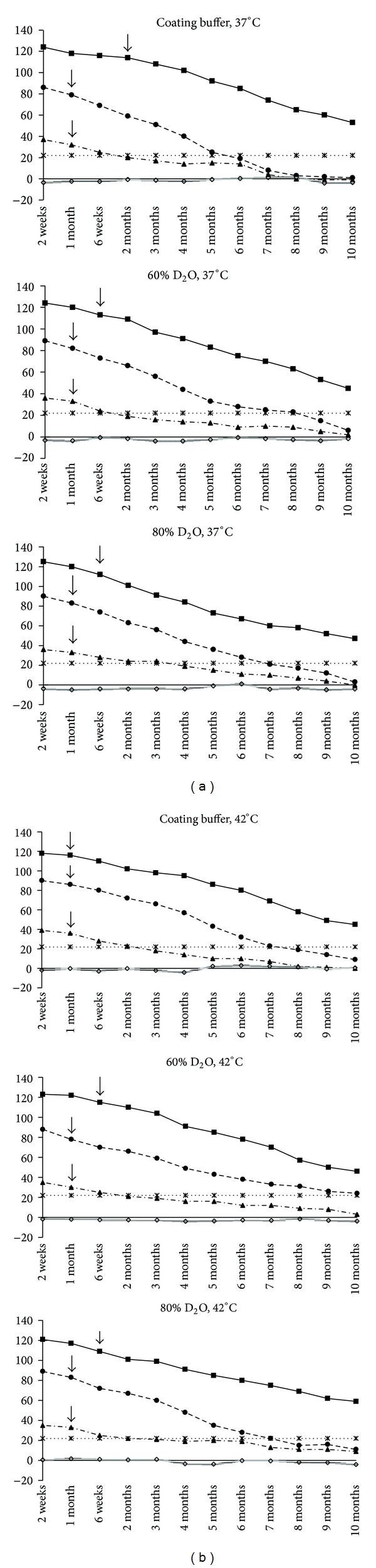
Thermostability of rp26 ELISA at 37°C (a) and at 42°C (b). Duration of thermostability is indicated by arrow (↓) against each strong serum positive serum (■), medium positive serum (●), and weak positive serum (▲). No significant difference exists in 60% D_2_O versus 80% D_2_O.

At 37°C in H_2_O coating buffer, *PP* value of strong positive serum significantly decreased from 114 at 2 months (*P* = 0.06) to 53 at 10 months (*P* = 0.005) while the *PP* value for medium and weak positive serums touched the cut-off point at 5 months and 6 weeks, respectively. In 60% and 80% D_2_O coating buffer, strong positive serum *PP* values fall from 113 at 6 weeks to 41 at 10 months. Like H_2_O coating buffer, the *PP* value for medium and weak positive serums touched the cut-off point at 6 months and 6 weeks, respectively, in D_2_O coating buffer at 37°C (Figure 2(a)).

At 42°C in H_2_O coating buffer, *PP* value of strong positive serum significantly decreased from 116 at 1 month (*P* = 0.06) to 45 at 10 months (*P* = 0.007) while the *PP* value for medium and weak positive serum touched the cut-off point at 7 months and 2 months, respectively. In 60% and 80% D_2_O coating buffer, strong positive serum *PP* values significantly fall from 115 and 111 at 6 weeks (*P* = 0.13, 0.06) to 46 and 59 at 10 months (*P* = 0.001, 0.007), respectively. The *PP* value for medium and weak positive serums touched the cut-off point at 9 months and 6 weeks, respectively, in 60% D_2_O coating buffer at 42°C (Figure 2(b)). In 80% D_2_O, the *PP* value touched the cut-off point at 7 months and 2 months for medium and weak positive serums, respectively.

**Figure 3:**
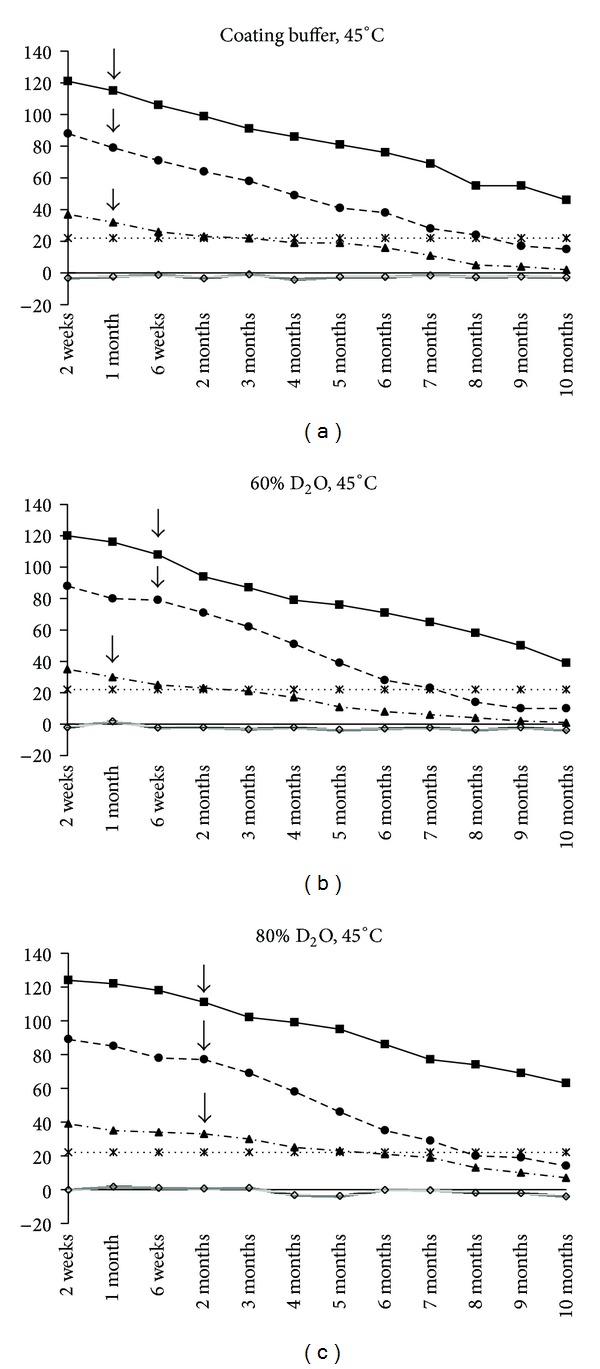
Thermostability of rp26 ELISA at 45°C. Duration of thermostability is indicated by arrow (↓) against strong positive serum (■), medium positive serum (●), and weak positive serum (▲). In 45°C, significant difference exists in 80% D_2_O versus 60% D_2_O, *P* = 0.01 − 0.001, 80% D_2_O versus H_2_O coating buffer, *P* = 0.02 − 0.001, at 2-month storage with respective serum strength.

In H_2_O coating buffer, no significant difference in *PP* value of strong, medium, and weak positive serums (115, 79, and 32, resp.) at 1 month was observed between 45°C and 42°C. The *PP* value of strong and medium positive serums (111 and 80 at 6 weeks) was slightly but significantly stable in 60% D_2_O than in the H_2_O coating buffer at 45°C (Figure 3). The *PP* value of weak positive serum touched the cut-off point at 6 weeks in both H_2_O and 60% D_2_O buffers. *PP* values of strong, medium, and weak positive serums were 111, 78, and 33, respectively, at 2 months in 80% D_2_O at 45°C. The *PP* value of weak positive serum touched the cut-off point at 4 months in 80% D_2_O buffer.

## 4. Discussion

Heavy water, formally called deuterium oxide or D_2_O, is a form of water that contains hydrogen isotope deuterium (D) rather than the common hydrogen-1 isotope (H) that makes up most of the hydrogen in normal water (H_2_O). The effects of D_2_O on living systems or biological macromolecules are exerted in two ways. The first one is “solvent isotope effect” which acts on the structure of water and the biological macromolecules. The other one is “deuterium isotope effect” where D_2_O replaces H with D in biological molecules [6]. The C–D bond is several times stronger than the C–H bond and thus more resistant to enzymatic and even chemical cleavage. This property has been utilized to enhance the thermostability of oral polio vaccine [9, 19], which shows D_2_O reconstituted vaccine and remains biologically active even if the cold chain is disturbed for a while. Later on, the same strategy has been adopted for stabilization of influenza vaccine [12], PPR vaccine [13], and haemorrahagic septicemia vaccine [14].

This paper describes for the first time *in situ* thermostabilization of equine infectious anemia virus (EIAV) recombinant p26 protein based indirect ELISA using D_2_O. Stabilizing effect was determined as a function of maintenance of statistically significant *PP* value over a storage time and temperature in comparison to freshly coated rp26 plate. It is important to mention that any diagnostic ELISA should be validated with varying strength of known positive serum to cover the broader range of diagnostic capacity, to improve the accuracy of diagnosis, or to avoid false negative findings. Therefore, in the present study, we have included three categories of positive serum differing in titer strength (strong, medium, and weak). It has been demonstrated that *PP* value of rp26 ELISA varies with serum strength (antibody titer), storage time, incubation temperature, and stability of protein in higher temperature. Although the *PP* values of strong, medium, and weak positive serums were above the cutoff at a given point, there was a significant deviation of the values in comparison to those in freshly coated plates. Therefore, to determine the actual duration of thermostability without compromising the overall diagnostic efficiency of ELISA, weak positive serum showing *PP* value equal to or statistically non-significantly different from that of fresh plate at given time point was considered.

Analysis of results demonstrated that rp26 in H_2_O or 60%, and 80% D_2_O based coating buffer was very stable at 4°C up to 5 months. Further, heavy water did not appear to have any stabilizing effect on protein at freezing temperature which is corroborated by earlier experiment in influenza vaccine [12]. It appears that it is possible to store rp26 coated and dried ELISA plate at 4°C up to 5 months and it could be used for diagnostic purpose. Drastic reduction in rp26 stability corresponding with steep decrease in *PP* value was observed at 37°C. There was no clear association between percentage of D_2_O and thermostable effect on ELISA at 37°C. D_2_O-based coating buffer was able to maintain diagnostically significant *PP* value up to 6 weeks for strong positive serum and 1 month for medium positive and weak positive serums (Figure 1).

Time- and temperature-dependent decrease in *PP* values is accelerated at higher temperature (42°C and 45°C) in the presence in of H_2_O-based coating buffer than in D_2_O-based coating buffer. The *PP* value of all positive serum significantly declined (*P* < 0.05) after 1 month in H_2_O coated plate. The rp26 protein yields diagnostically comparable *PP* value with strong positive serum till 6 weeks at 42°C in D_2_O coating buffer (Figure 3). However, for medium and weak positive serum protective effect of D_2_O on rp26 protein was observed till 1 month at 42°C. Again, concentration of D_2_O had no significant effect on rendering thermal protection to rp26 protein at 42°C. The greatest degree of stabilization was obtained with the highest concentration (80%) of D_2_O at 45°C. The 80% D_2_O provides the thermal protection to rp26 protein in ELISA plate up to 2 months of incubation at 45°C. Among the three coating buffers, 80% D_2_O buffer was found to have the most thermostable effect at 45°C, followed by 60% D_2_O as compared to H_2_O.

No difference in stability of rp26 protein between 42°C and 45°C in H_2_O coating buffer was found. However, thermal tolerance of rp26 protein at elevated temperature for a month in H_2_O-based coating buffer suggests that rp26 protein could remain stable at 42°C or 45°C up to certain period (1 month), whenever 80% D_2_O enhances the thermal protection to rp26 protein up to 2 months at higher temperature (45°C). The protective effect of the 80% D_2_O was more evident at 45°C temperature than that of 60% D_2_O. Gradual increase in the stabilizing effect of 80% D_2_O at elevated temperature (37°C < 42°C < 45°C) observed in the present study correlates with previous findings where ~90% deuterium oxide results in a significant increase in the stability of the polio and influenza virus incubated at 37°C, 42°C, 45°C, or 56°C [8, 12]. To elucidate the mechanism of action of D_2_O on stabilizing, the recombinant protein was beyond the scope of the study. However, it may be inferred from the earlier investigations that D_2_O may have rendered the protective effects to rp26 by providing structural rigidity to protein molecules due to the greater strength of noncovalent oxygen-deuterium or nitrogen-deuterium bonds relative to the corresponding hydrogen bonds resulting in slowing down of degradative reaction [10, 12, 19, 20].

This strategy of stabilizing protein using D_2_O seems to be applicable to a wide range of assays involving recombinant proteins. The utility of this strategy may be limited at present; however, this is a step forward towards developing a thermostable immunoassay suitable for tropical countries and can serve as an alternative support system for cold chain. In the present study, rp26 was exposed to D_2_O only for 16 h; long-term exposure to D_2_O might have better thermostable effect which needs further verification. The findings of the present study have the future implication of adopting cost effective strategies for generating more heat tolerable ELISA reagents with extended shelf life.
